# Fabrication of Li_4_Ti_5_O_12_@CN Composite With Enhanced Rate Properties

**DOI:** 10.3389/fchem.2019.00432

**Published:** 2019-06-14

**Authors:** Hui Xiao, Xiaobing Huang, Yurong Ren, Xiang Ding, Shibiao Zhou

**Affiliations:** ^1^School of Materials Science and Engineering, Jiangsu Collaborative Innovation Center of Photovolatic Science and Engineering, Changzhou University, Changzhou, China; ^2^Hunan Province Cooperative Innovation Center for The Construction and Development of Dongting Lake Ecological Economic Zone, College of Chemistry and Materials Engineering, Hunan University of Arts and Science, Changde, China

**Keywords:** Li-ion batteries, anode material, folic acid, Li_4_Ti_5_O_12_, N-doped carbon coating

## Abstract

Folic acid is first time applied as a carbon-nitrogen precursor to fabricate Li_4_Ti_5_O_12_@CN composites via ball milling Nano-TiO_2_, Li_2_CO_3_ and folic acid with ethanol as solvent, and then followed by heating treatment in argon. XRD, SEM, TEM, XPS, charge-discharge test and EIS are used to evaluate the influence of N-doped carbon coating on its structure, morphologies and electrochemical property. It is demonstrated that the N-doped carbon coated Li_4_Ti_5_O_12_ composite exhibits superior high-rate performance compared with pure Li_4_Ti_5_O_12_. It possesses a high discharge capacity of 174, 165 mAh g^−1^ at 0.5 and 10 C, respectively. Additionally, an initial specific capacity of 96.2% is obtained after 200 cycles at 10 C. The remarkable performance might be put down to the N-doped carbon layer providing efficiently electron conductive network and nanosized decreasing lithium ion diffusion path.

## Introduction

In the past few years, carbon materials have been regarded as the most commercially valued lithium battery anode material (Ma et al., 2016; Long et al., 2017; Li et al., 2019; Wu et al., 2019). Unfortunately, its low lithiation potential (~0.2 V vs. Li^+^/Li) will result in the activation of dendritic lithium, thus further creating safety problems. Moreover, insufficient diffusion coefficient of lithium ion and large volume change will result in poor rate performance and cycle stability (Han et al., 2018; Yi et al., 2018). Therefore, it is essential to find alternative anodes with excellent electrochemical properties and outstanding safety characteristics. Among the reported alternative candidates, Spinel lithium titanium (Li_4_Ti_5_O_12_) is suggested as a potential anode material used in lithium-ion batteries (LIBs) due to the following reasons: (i) The higher flat discharge and charge plateau (1.55 V vs. Li^+^/Li) can prevent lithium metal dendrites from evolving during the electrochemical reaction process (Wang S. et al., 2017). (ii) Small volume expansion provides superior cycle stability and reversibility among charge and discharge process (Tian et al., 2017; Wang Q. et al., 2017). Nevertheless, its small lithium ion diffusion coefficients (10^−9^ ~10^−13^ cm^2^ S^−1^) and weak electrical conductivity (~10^−13^ S cm^−1^) (Jiang et al., 2017), resulting in serious electrode polarization and poor capability at high current density, has been considered as the main bottleneck of its commercial application for high-power LIBs. In order to overcover this problem, several strategies have been suggested by researchers, including synthesis of Li_4_Ti_5_O_12_ with porous structure (Lu et al., 2017), coating the surface of Li_4_Ti_5_O_12_ particles with conductive materials (Tang et al., 2017), construction of nanoscale particle size (Chiu et al., 2017), and introduction of metal and nonmetal ion into Li_4_Ti_5_O_12_ (Chen et al., 2017; Cheng et al., 2017;Liang et al., 2017).

Thin carbon coating on the surface of Li_4_Ti_5_O_12_ particles has been considered as an effective method to enhance its electrochemical properties in all reported literatures, since the carbon coating can both improve the surface electron conductivity and inhibit the growth of primary particles in the heat treatment process, this further leads to faster lithium ion diffusion. Very recently, the introduction of nitrogen-doped carbon to modify the electrode materials has been received more and more attention. It is well-known that N atoms can provide additional electrons to further increase the conductivity of the coated carbon layer (Jiang et al., 2017; Xu et al., 2017). In addition, N-doped is favorable to reduce the barrier of energy of lithium-ion penetration and enhance reaction sites (Xiong et al., 2018). We have just proposed folic acid as a new carbon nitrogen precursor to prepare Na_3_V_2_(PO_4_)_3_@CN composite material, it possesses excellent rate performance and excellent cycling performance when used as cathode electrode of sodium ion batteries. In this work, a similar strategy was suggested to prepared Li_4_Ti_5_O_12_@CN composite. It is well-expected that the as-prepared Li_4_Ti_5_O_12_@CN sample will possess excellent electrochemical characters.

## Experimental

### Material Preparation

Li_4_Ti_5_O_12_@CN composite was synthesized via a solid-state process, and the detail process was described as following. Firstly, 2.2281 g of Li_2_CO_3_ (99.5%),5.9985 g of TiO_2_ (99.9%) and 1 g of folic acid were mixed by ball milling in ethyl alcohol for 6 h. Secondly, the solvent was evaporated by drying the ready-prepared mixture at 80°C for 2 h. Thirdly, the synthesized precursor was heated under argon atmosphere at 750°C for 8 h. For comparison, pure Li_4_Ti_5_O_12_ was also synthesized via the similar method without using folic acid as the starting material.

### Characterization of Materials

X-ray diffraction instrument was used to investigate the structure and composition of both as-prepared Li_4_Ti_5_O_12_ samples. The surface chemical states of Li_4_Ti_5_O_12_@CN were identified by XPS. SEM was used for observing morphologies of the two ready-prepared Li_4_Ti_5_O_12_ samples. N-doped carbon layer was further investigated by TEM. Elemental analyzer was carried out to investigate the content of carbon and nitrogen for Li_4_Ti_5_O_12_@CN composite. Four-point probe method was used to investigate the electronic conductivities of both Li_4_Ti_5_O_12_ samples.

### Electrochemical Measurements

The fabricated working electrodes consisted of as-synthesized Li_4_Ti_5_O_12_ sample, LA-132 and Super-P in a weight ratio of 85:5:10. The mixture was uniformly casted onto the aluminum foil and then dried in vacuum. CR2032 coin type cells were prepared in glove box with filled argon by composing of lithium piece as the counter electrode, 1 mol/L LiPF_6_ in EC/DEC/DMC (1:1:1 in volume) as the electrolyte. Celgard 2400 as the separator,. LAND CT2001 system were used to investigate the charge-discharge experiments between the potential range of 1–3V. Electrochemical impedance spectra (EIS) were investigated by using CHI600E electrochemical station in a frequency range of 10^−2^−10^5^ Hz.

## Results and Discussion

**Figure 1:**
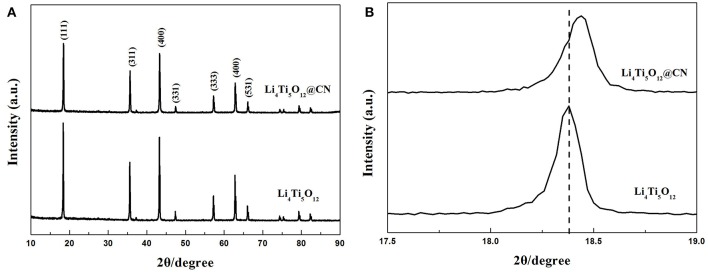
**(A)** X-ray diffraction profiles of Li_4_Ti_5_O_12_ and Li_4_Ti_5_O_12_@CN composite, **(B)** Enlarged (111) peak of Li_4_Ti_5_O_12_ and Li_4_Ti_5_O_12_@CN composite.

Crystal structure of both as-obtained Li_4_Ti_5_O_12_ samples was characterized via XRD, and the results are plotted in Figure 1. It clearly verifies that the dominating diffraction peaks of both as-synthesized Li_4_Ti_5_O_12_ samples are in consistence with the base peaks of spinel Li_4_Ti_5_O_12_ (PDF No.49-0207), demonstrating that the nitrogen-doped carbon layer coating process has little effect on the formation of spinel Li_4_Ti_5_O_12_ (Li et al., 2013a; Chang et al., 2014; Wang P. et al., 2017). Perhaps due to the amorphous morphology of carbon, the diffraction peak relative to carbon was not observed (Xu et al., 2017; Liu et al., 2018). The detail of the enlarged peak corresponding to Li_4_Ti_5_O_12_ (111) plane was described in Figure 1B. As clearly found that the central position of this peak shifts to larger angles after N-doped carbon coating, suggesting that nitrogen atoms possibly doped into the Li_4_Ti_5_O_12_ lattice and form a new thin layer of TiN_x_, similar phenomenon was also reported by Li et al. (Zhang et al., 2013). In addition, the intensity of the peaks in Li_4_Ti_5_O_12_@CN composite is lower than that of pure Li_4_Ti_5_O_12_, indicating that the N-doped carbon coating on the surface of Li_4_Ti_5_O_12_ will prevent the growth of particles. In order to investigate the content of carbon and nitrogen in Li_4_Ti_5_O_12_@CN composite, elemental analysis measurement is carried out. The content of carbon and nitrogen for Li_4_Ti_5_O_12_@CN composite is 1.46 and 0.24%, respectively.

**Figure 2:**
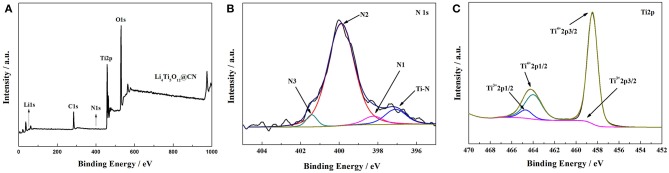
**(A)** X-ray photoelectron spectroscopy survey spectra of Li_4_Ti_5_O_12_@CN composite, **(B)** high-resolution XPS spectra of N1s, **(C)** high-resolution XPS spectra of Ti2p.

The obtained results of surface chemical state of Li_4_Ti_5_O_12_@CN composite evaluated by X-ray photoelectron spectroscopy (XPS) are given in Figure 2. Li1s, Ti2p, O1s, C1s, and N1s peaks are observed from the XPS spectra of Li_4_Ti_5_O_12_@CN composite (Figure 2A). The high-resolution N1s of Li_4_Ti_5_O_12_@CN composite is demonstrated in Figure 2B. As illustrated in Figure 2B, pyridine (N_1_), pyrrole (N_2_) and graphitic (N_3_) correspond to peaks centered at 398.2, 399.8, and 401.4eV, respectively. (Li H. et al., 2014; Long et al., 2015; Wang et al., 2015). The above results clearly showed that nitrogen-doping is successfully introduced in the carbon layer by using folic acid as a carbon-nitrogen precursor, which might result in produce the flaws in the symmetric offset spread of aromatic rings carbon, and thus further increase the diffusion of Li^+^ in the interface (Li et al., 2013a; Wang et al., 2015). Additionally, a peak at about 397 eV being attributed to the interaction energy of TiN is observed in Figure 2B, indicating that titanium nitride (TiN) phase is created during the sintering process (Wan et al., 2012). As well-accepted, the existence of TiN with a metallic conductivity will improve electronic conductivity (Li H. et al., 2014). The high-resolution Ti2p of Li_4_Ti_5_O_12_@CN composite is depicted in Figure 2C. Clearly, two peaks appeared at approximately 464.2 and 458.5eV are observed, which represents to peaks of Ti 2p1/2 and Ti 2p3/2 of Ti^4+^ in the sample (Li et al., 2014). In addition, two extra peaks located at about 459 and 464.7eV are detected, corresponding to the peaks of Ti 2p1/2 and Ti 2p3/2 of Ti^3+^ in the sample, respectively, which suggests that Ti^3+^ sites were introduced in the Li_4_Ti_5_O_12_@CN composite due to the reduced ability of N-doped carbon. Similar phenomena were also demonstrated in previous reports (Wan et al., 2012; Ming et al., 2014). It further verified that the titanium nitride (TiN) phase was formed.

**Figure 3:**
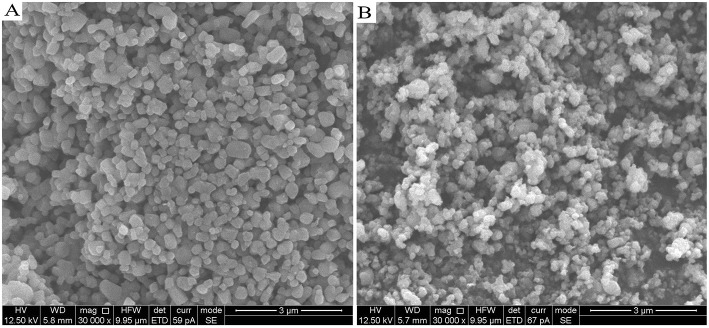
SEM pictures of Li_4_Ti_5_O_12_ and Li_4_Ti_5_O_12_@CN composite.

**Figure 4:**
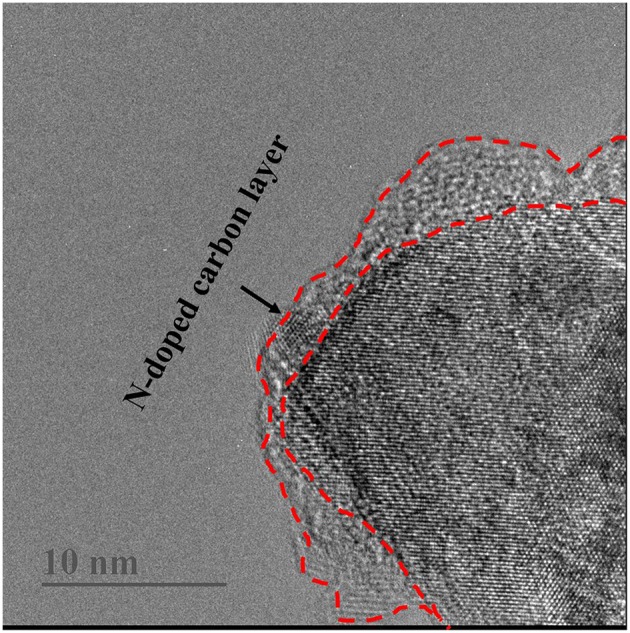
TEM image of Li_4_Ti_5_O_12_@CN composite.

The different morphology and particle size between Li_4_Ti_5_O_12_ and Li_4_Ti_5_O_12_@CN composites were investigated by SEM characterization. The results are shown in Figure 3. As clearly demonstrated in Figure 3, Li_4_Ti_5_O_12_@CN composite possesses a much smaller particle size than that of Li_4_Ti_5_O_12_. It is well-accepted that *in situ* N-doped carbon coating will well-prohibit the Li_4_Ti_5_O_12_ particles from growing into larger crystals (Wang C. et al., 2014). The SEM results agree well with the XRD results. To further verify this prediction, the specific surface area of both as-obtained Li_4_Ti_5_O_12_ samples was investigated. Li_4_Ti_5_O_12_@CN composite and pure Li_4_Ti_5_O_12_ possess a specific surface area of 12.75 and 7.08 m^2^ g^−1^, respectively. Generally, much smaller particles of Li_4_Ti_5_O_12_@CN composite gives the larger specific surface area. As well-accepted that fabrication of material with much smaller particle size and larger specific surface area will decrease the lithium ion diffusion pathways and thus enhance the kinetics of lithiation/delithiation (Long et al., 2015). The carbon-nitrogen layer of Li_4_Ti_5_O_12_@CN composite was further studied by TEM characterization and results are demonstrated in Figure 4. It was found that a carbon-nitrogen layer with a thickness of 2 to 5 nm is formed on the exterior of Li_4_Ti_5_O_12_. The electronic conductivities of the as-synthesized Li_4_Ti_5_O_12_ samples were confirmed by four-point probe method. Pure Li_4_Ti_5_O_12_ and Li_4_Ti_5_O_12_@CN composite have the electronic conductivities of 7.67 × 10^−5^ and 1.06 × 10^−2^ S cm^−1^, respectively.

**Figure 5:**
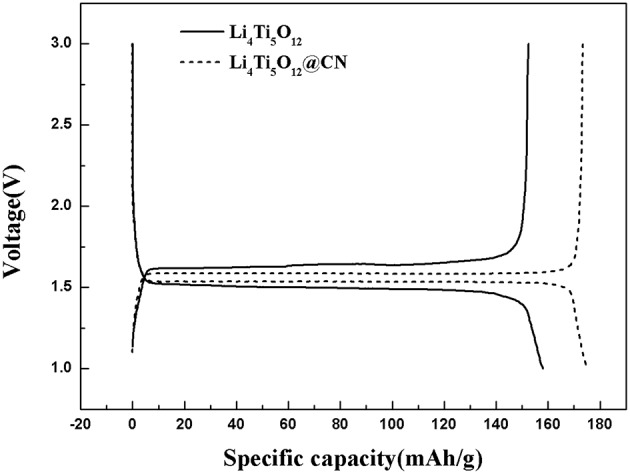
The first charge-discharge cycles for Li_4_Ti_5_O_12_ and Li_4_Ti_5_O_12_@CN composite at 0.2 C.

The first charge-discharge cycle for as-synthesized Li_4_Ti_5_O_12_ and Li_4_Ti_5_O_12_@CN composite at a rate of 0.5 C between a voltage range of 1–3V are described in Figure 5. Both as-prepared Li_4_Ti_5_O_12_ samples possess a voltage plateau at ~1.55V, suggesting a two-phase reaction is carried out on the basis of the redox pair of Ti^3+^/Ti^4+^ (Wang et al., 2012; Wang B. et al., 2014). However, the potential separation between the charge and discharge curves of Li_4_Ti_5_O_12_@CN composite material is much smaller than that of Li_4_Ti_5_O_12_, indicating that the Li_4_Ti_5_O_12_@CN electrode has less polarization and better reaction kinetics, which demonstrates that the enhancement of electrical conductivity of Li_4_Ti_5_O_12_ after introducing N-doped carbon coating (Li et al., 2013a; Zhang et al., 2014).

**Figure 6:**
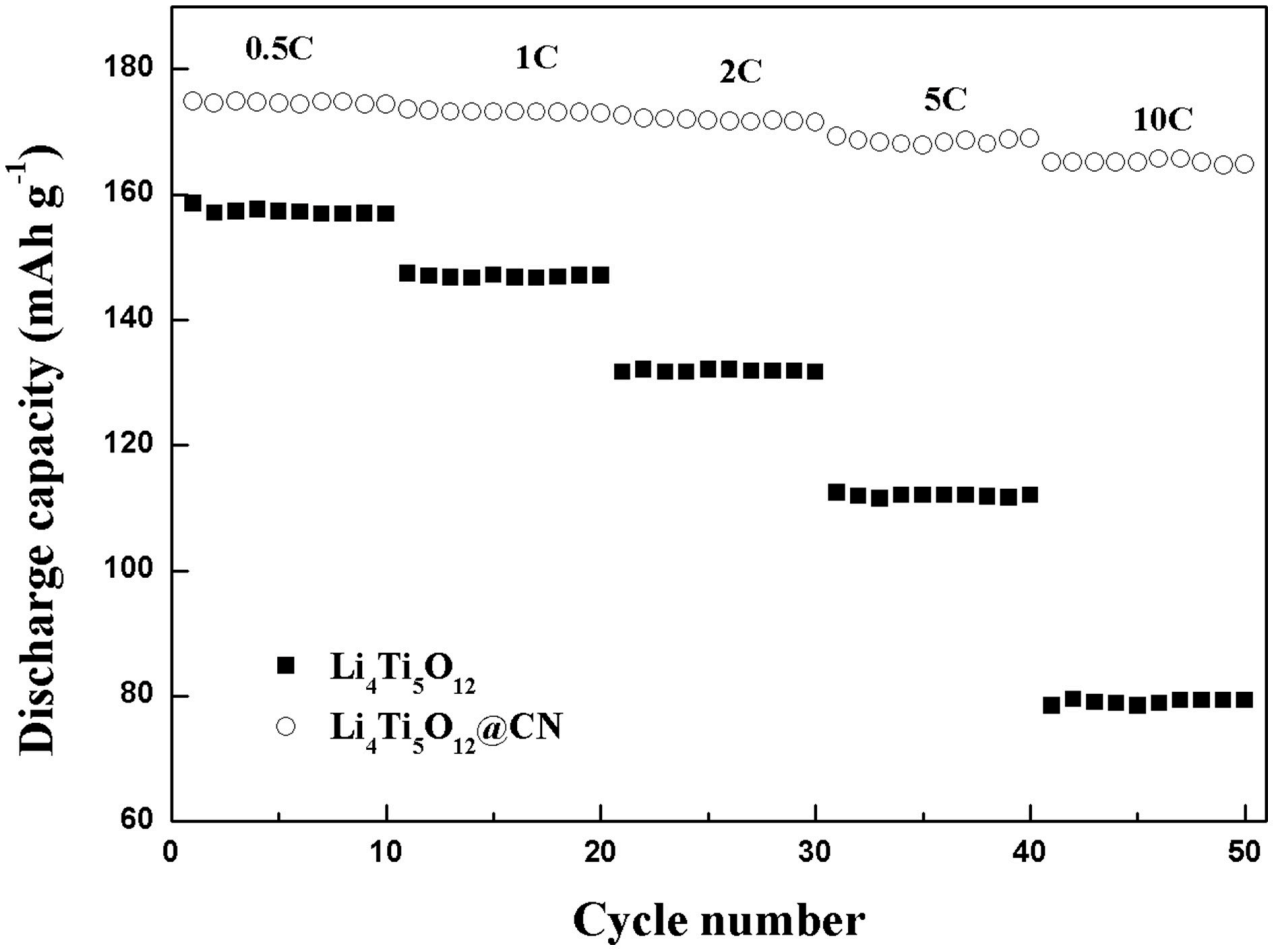
Rate property of Li_4_Ti_5_O_12_ and Li_4_Ti_5_O_12_@CN composite at different rates from 0.5 C to10 C.

The rate capabilities of Li_4_Ti_5_O_12_@CN composite and Li_4_Ti_5_O_12_ are shown in Figure 6. Pure Li_4_Ti_5_O_12_ obtained a discharge capacity of 158 mAh g^−1^ at a low rate of 0.5 C, and the capacity decreases remarkably as the rate increased from 0.5 to 1, 2, 5, and 10 C, respectively. Especially, its discharge capacity is only 79 mAh g^−1^ at 10 C. The poor rate properties of pure Li_4_Ti_5_O_12_ could be due to its poor conductivity. While much-improved discharge capacity at each rate for Li_4_Ti_5_O_12_@CN composite in comparison with pure Li_4_Ti_5_O_12_. At 0.5, 5, and 10 C, its discharge capacity were 174, 168, and 165 mAh g^−1^, respectively. The superior rate properties of Li_4_Ti_5_O_12_@CN composite could be due to three reasons: (i) As demonstrated in SEM results (Seen in Figure 4), the smaller particle size for Li_4_Ti_5_O_12_@CN composite is favorable for the faster Li^+^ diffusion and further enhancement of kinetic coefficient of lithium ion embedded into Li_4_Ti_5_O_12_ structure (Long et al., 2015). (ii) The electronic conductivity of Li_4_Ti_5_O_12_ is deemed to be enhanced by N-doped carbon coating (Xu et al., 2017), and the defects in the carbon layer caused by N-doping facilitate Li^+^ migration in the interface (Li et al., 2013b). (iii) The electronic conductivity properties of Li_4_Ti_5_O_12_ will be further increased with the existence of TiN_x_ in the composite.

**Figure 7:**
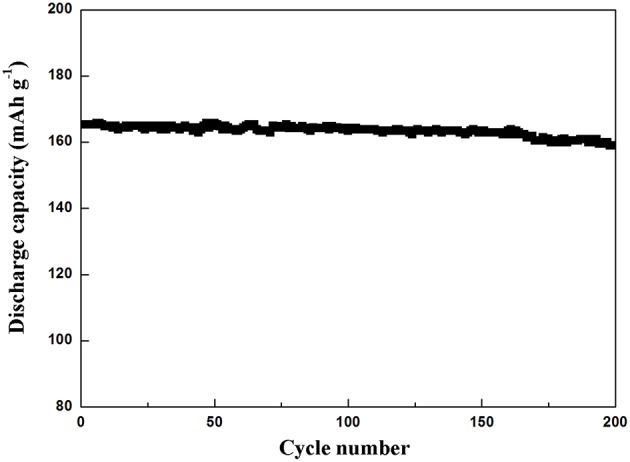
Cycle property of Li_4_Ti_5_O_12_@CN composite at 10 C.

The cyclic property of Li_4_Ti_5_O_12_@CN composite at a rate of 10 C is depicted in Figure 7. As clearly seen, the initial discharge capacity of Li_4_Ti_5_O_12_@CN composite is 165 mAh g^−1^, and 96.2% of its capacity is obtained after 200 cycles, suggesting that the as-synthesized Li_4_Ti_5_O_12_@CN composite possesses good cycle stability.

**Figure 8:**
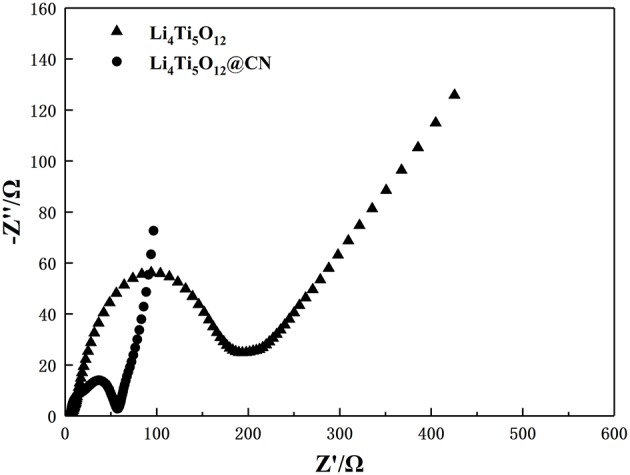
Nyquist plots of Li_4_Ti_5_O_12_ and Li_4_Ti_5_O_12_@CN composite.

To further investigate the effect of N-doped carbon coating on electrode behavior, electrochemical impedance spectroscopy measurements were performed on Li_4_Ti_5_O_12_ and Li_4_Ti_5_O_12_@CN electrodes, and the results are plotted in Figure 8. Obviously, each electrode exhibits a similar profile with a straight line at the low frequency region and a depressed semicircle at the high-middle frequency range, being correlated with Li-ion diffusion and charges transfer resistance (Rct) in the electrode/electrolyte interface, respectively (Zheng et al., 2018; An et al., 2019; He et al., 2019; Lv et al., 2019; Xiao et al., 2019; Zhou et al., 2019). As obtained from Figure 8, Li_4_Ti_5_O_12_@CN electrode exhibits much smaller charge-transfer resistance of 52 Ω than that of 194 Ω for pure Li_4_Ti_5_O_12_ electrode, suggesting the improved electronic conductivity of Li_4_Ti_5_O_12_@CN composite in comparison with pure Li_4_Ti_5_O_12_ caused by the highly electronic conductive coating with N-doped carbon as well as the presence of TiN_x_ phase (Zhou et al., 2017). According to the following equation, the Li-ion diffusion coefficient of both as-prepared Li_4_Ti_5_O_12_ samples can be obtained:

(1) $$\text{D}={\text{R}}^{\text{2}}{\text{T}}^{\text{2}}{\text{/2A}}^{\text{2}}{\text{n}}^{\text{4}}{\text{F}}^{\text{4}}{\text{C}}^{\text{2}}\sigma^{\text{2}}$$

(2) $${\text{Z}}_{\text{re}}={\text{R}}_{\text{D}}+{\text{R}}_{\text{L}}+{\sigma \omega }^{-\text{1/2}}$$

**Figure 9:**
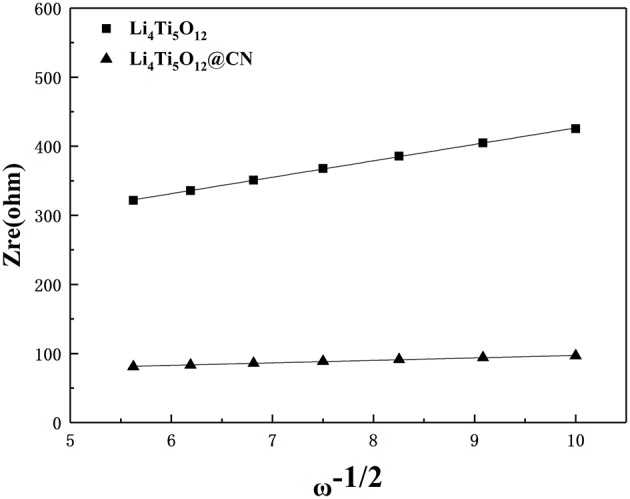
Relation Z_re_ and ω^−1/2^ in between at the low frequency region.

The value of σ for both samples could be obtained from the lines described in Figure 9. Based on the above equations and the results from Figure 9, the obtained lithium ion diffusion coefficient for Li_4_Ti_5_O_12_ and Li_4_Ti_5_O_12_@CN composite is 6.58 × 10^−11^ and 2.89 × 10^−9^ cm^2^ s^−1^, respectively. There is no doubt that the Li_4_Ti_5_O_12_@CN electrode has a larger Li-ion diffusion coefficient due to the smaller particle size and the carbon layer defect of the Li_4_Ti_5_O_12_@CN composite, indicating that a valid enhancement of the diffusion kinetics of Li_4_Ti_5_O_12_ after the introduction of the N-doped carbon coating. Based on the above results and discussion, coating with N-doped carbon can dramatically improve the lithium ion and conductive property. Consequently, Li_4_Ti_5_O_12_@CN composite has much improved electrochemical performance in compassion with pure Li_4_Ti_5_O_12_.

## Conclusions

In this study, N-doped carbon-coated Li_4_Ti_5_O_12_ is prepared with folic acid as a carbon-nitrogen source. Li_4_Ti_5_O_12_@CN composite has the outstanding reversible capacity, high rate capability in compassion with pure Li_4_Ti_5_O_12_. The discharge capacity of the Li_4_Ti_5_O_12_@CN composite at 10 C was 165 mAh g^−1^, and the initial specific capacity remained at 96.2% after 200 cycles. The superior properties of Li_4_Ti_5_O_12_@CN composite could be owing to the improved electronic conductivity caused by the N-doped carbon layer and the TiN phase as well as enhanced Li-ion diffusion coefficient rising from the smaller particle size and the defects in the carbon layer.

## Data Availability

The raw data supporting the conclusions of this manuscript will be made available by the authors, without undue reservation, to any qualified researcher.

## Author Contributions

XH and YR contributed conception and design of the study. HX carried out experiments and wrote the manuscript. XD performed analyzed experimental results. SZ revised the manuscript.

### Conflict of Interest Statement

The authors declare that the research was conducted in the absence of any commercial or financial relationships that could be construed as a potential conflict of interest.
